# Phase junction enhanced photocatalytic activity of Ga_2_O_3_ nanorod arrays on flexible glass fiber fabric†

**DOI:** 10.1039/d0ra01461c

**Published:** 2020-03-20

**Authors:** Hanlin Sun, Liying Zhang, Jingyan Yu, Shunli Wang, Daoyou Guo, Chaorong Li, Fengmin Wu, Aiping Liu, Peigang Li, Weihua Tang

**Affiliations:** Key Laboratory of Optical Field Manipulation of Zhejiang Province, Center for Optoelectronics Materials and Devices, Department of Physics, Zhejiang Sci-Tech University Hangzhou 310018 China slwang@zstu.edu.cn; State Key Laboratory of Information Photonics and Optical Communications, Information Functional Materials and Devices, School of Science, Beijing University of Posts and Telecommunications Beijing 100876 China

## Abstract

Ga_2_O_3_ nanostructures hold great potential applications in photocatalytic fields due to their stability, high efficiency and environmental friendliness. The construction of phase junction has been proved to be one of the most effective strategies for enhancing Ga_2_O_3_ photocatalytic activity. However, the influence of the formation process at the interface of the phase junction on the photocatalytic activity of Ga_2_O_3_ nanostructures is far less well understood. In this work, for the first time, large-area Ga_2_O_3_ nanorod arrays (NRAs) with controllable α/β phase junction were prepared *in situ* on a flexible glass fiber fabric by a facile and environmentally friendly three-step method. The α/β-Ga_2_O_3_ phase junction NRAs exhibit an ultra-high photocatalytic degradation rate of 97% during Ultraviolet (UV) irradiation for 60 min, which is attributed to a unique phase junction promoting efficient charge separation. However, the photocatalytic activity of α/β-Ga_2_O_3_ phase junction NRAs is not evident in the early phase transition, possibly due to the presence of defects acting as charge recombination centers.

## Abstract 2

Ga_2_O_3_ nanostructures hold great potential applications in photocatalytic fields due to their stability, high efficiency and environmental friendliness.

## Graphical abstract

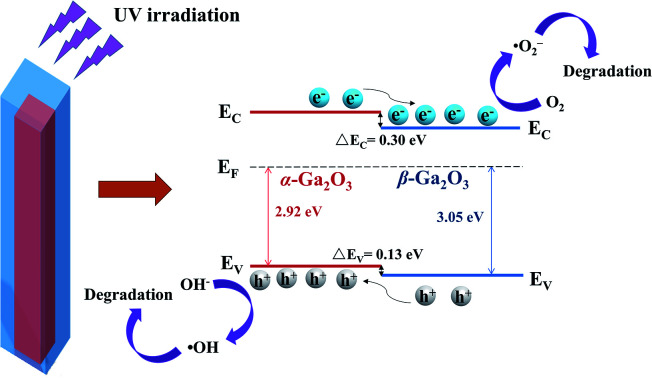

## 1. Introduction

With the continuous development of the social economy, environmental pollution has become an increasingly serious problem, prompting humans to continuously explore new solutions.^1–6^ Photocatalytic reaction, as a simple, efficient and cost-effective method, has promising applications in the removal of environmental pollutants and attracted wide attention.^7–13^ Recently, various metal oxides with d^10^ (In^3+^, Ga^3+^, Ge^4+^, Sn^4+^) configurations have been reported as effective photocatalysts for photodegradation of various organic pollutants.^14,15^ Ga_2_O_3_ is a typical representative among them.^16^

With a wide bandgap (4.2–4.9 eV) and excellent physical and chemical properties, Ga_2_O_3_ is recognized as one of the most promising semiconductors of this century.^17–22^ It has extensively been applied to power devices,^23–25^ solar-blind ultraviolet (UV) photodetectors,^26–29^ gas sensors,^30^ solar cells^31^ and photocatalysis.^32,33^ For photocatalysis applications, related studies claim that Ga_2_O_3_ can theoretically exhibit better and more stable photocatalytic activity than commercial TiO_2_ and realize the degradation of refractory pollutants.^34,35^ This is attributed to the extraordinary redox capability of photogenerated electron–hole pairs.^1,36,37^ Furthermore, Ga_2_O_3_ is also widely accepted as an environmentally friendly material with low cost and high chemical stability.^38^ Many methods have been investigated to further improve the photocatalytic activity of Ga_2_O_3_, including morphology controlling, doping, surface modification and semiconductor coupling.^39–44^ Nitu Syed *et al.* reported a two-step method for the synthesis of porous α-Ga_2_O_3_ nanosheets from liquid metal gallium, explaining that the excellent photocatalytic activity of α-Ga_2_O_3_ originated from the narrowed bandgap caused by trap states.^1^ Han *et al.* revealed that the modification of *in situ* Ag nanoparticles can effectively improve the photocatalytic property of Ga_2_O_3_ for hydrogen evolution.^39^ Zhang *et al.* incorporated solvothermally synthesized Ga_2_O_3_ nanoparticles into liquid metal/metal oxide frameworks to form enhanced photocatalytic systems.^41^ Xu *et al.* fabricated two-dimensional TiO_2_–Ga_2_O_3_ p–n heterostructures, demonstrating the contribution of heterostructures in enhancing photocatalytic activity.^45^

Furthermore, the construction of appropriate phase junction structure in Ga_2_O_3_ can also significantly enhance photocatalytic activity.^46–48^ Liu *et al.* demonstrated that the mesopores and heterojunction in the mixed-phase Ga_2_O_3_ are responsible for enhancing photocatalytic activity.^7^ However, the influence of the formation process at the interface of the phase junction on the photocatalytic activity of Ga_2_O_3_ nanostructures has not been fully understood. For example, phase transformation is a process from the surface to the bulk, and different thicknesses of phase interface may result in various photocatalytic activities.^49^ Therefore, an in-depth understanding of junction-related issues will aid in the design and preparation of efficient Ga_2_O_3_ nanostructured photocatalysts. On the other hand, almost all of the reported Ga_2_O_3_ nanostructured photocatalysts are currently applied in suspension systems. The disadvantages of photocatalysts, such as agglomeration, inadequate illumination and difficulty in recovery, restrict their large-scale practical applications. Glass fiber fabric as a support for *in situ* growth of Ga_2_O_3_ nanostructures is expected to effectively overcome this difficulty.^18^ To the best of our knowledge, there are no reports of *in situ* preparation of Ga_2_O_3_ nanostructures on glass fiber fabric for the application of photocatalytic degradation.

Herein, for the first time, we reported a facile and environmentally friendly three-step method for *in situ* preparation of large-area Ga_2_O_3_ nanorod arrays (NRAs) with controllable α/β phase junction on a flexible glass fiber fabric. The as-prepared α/β-Ga_2_O_3_ phase junction NRAs exhibited excellent photocatalytic activity for the degradation of Rhodamine B (RhB) aqueous solution. In addition, the mechanism of photocatalytic activity enhancement was discussed and compared with related literature.

## 2. Experimental

### 2.1. Materials

Glass fiber fabric, model TS-BXB, specification 0.06 MM × 1.20 M, obtained from Hangzhou Gaojing Fine Chemical Industry Co., Ltd. The glass fiber fabrics were cut into a size of approximately 20 × 20 mm^2^ as the substrate of Ga_2_O_3_ NRAs. Rhodamine B (RhB), gallium nitrate hydrate (Ga(NO_3_)_3_·*n*H_2_O) were purchased from Shanghai Saen Chemical Technology Co., Ltd. Fluorine doped tin oxide (FTO) conductive glass (14 Ω cm^−2^, size: 10 × 20 × 2.2 mm^3^) was made by Japan Nippon Sheet Glass Co., Ltd. Sodium sulphate (Na_2_SO_4_) was got from Tianjin Yongda Chemical Regent Co., Ltd. All chemicals are analytical grade.

### 2.2. Sample preparation

**Fig. 1:**
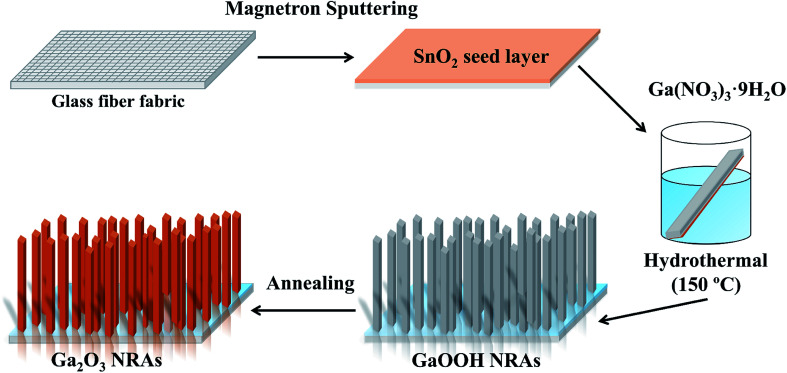
Schematic illustration of the preparation process of α-Ga_2_O_3_ NRAs.

The preparation of Ga_2_O_3_ NRAs involves the following three steps. In the first step, a SnO_2_ thin film was fabricated by radio frequency magnetron sputtering on the surface of the cleaned glass fiber fabric, which was used as a growth seed layer of Ga_2_O_3_. The growth temperature and Ar gas pressure were fixed at 550 °C and 0.8 Pa, respectively. The second step is to prepare GaOOH nanorod precursor by hydrothermal method. Here, 0.20 g of Ga(NO_3_)_3_·*n*H_2_O was dissolved in 30 mL of DI water to prepare a growth solution. Then the substrates glass fiber fabric completed in the first step was placed in the growth solution and transferred separately into a 50 mL Teflon-lined stainless steel autoclave for hydrothermal treatment at 150 °C for 12 h. After the solution was naturally cooled down to room temperature, the precipitates were filtered and washed with DI water, then dried in air at 80 °C for 2 h to obtain GaOOH NRAs precursors. The last step, the as-prepared precursors were annealed at 400 °C for 4 h in air to obtain α-Ga_2_O_3_ NRAs. The detailed synthesis is schematically demonstrated in Fig. 1. Further, other samples were obtained by annealing α-Ga_2_O_3_ NRAs in air at 700 °C for different times from 20 min to 120 min.

### 2.3. Characterization

The crystal structure of samples was analyzed by a Bruker D8 DISCOVER X-ray diffractometer (XRD). UV-Raman spectra were recorded on a Jobin-Yvon T64000 triple-stage spectrograph with spectral resolution of 2 cm^−1^. The thermal behavior of the GaOOH nanorod was investigated by thermal gravimetric analyzer (Pyris1 TGA). For the morphological and microstructural analysis, a Hitachi S-4800 field-emission scanning electron microscope (SEM) equipped and a JEOL JEM-2100 transmission electron microscopy (TEM) were utilized. The ultraviolet-visible (UV-vis) absorption spectra were taken using a Hitachi U-3900 UV-vis spectrophotometer. The chemical composition of samples was characterized by a Thermo Scientific K-Alpha X-ray photoelectron spectroscopy (XPS).

### 2.4. Photocatalytic experiments

In this experiment, the glass fiber fabric with Ga_2_O_3_ NRAs were dropped into 50 mL of RhB aqueous solution (2 × 10^−5^ M) and placed in the dark for 30 min to ensure adsorption–desorption equilibrium was reached. Then irradiated reaction solution with a 10 W UV light lamp (*λ* = 254 nm). The light intensity of the UV lamp was always maintained at 1.0 mW cm^−2^. During the process, about 3 mL of solution was withdrawn from the reaction system at a given time interval (10 min) for absorbance testing by UV-vis spectrophotometry.

### 2.5. Mott–Schottky measurement

For Mott–Schottky measurements, 5 mg Ga_2_O_3_ NRAs powder was scraped from the glass fiber fabric and dispersed in 2 mL of absolute ethanol, followed by the addition of 20 μL of 0.5% Nafion. After the mixed solution was sonicated for 1 h, 0.5 mL was transferred onto a FTO conductive glass. The resulting electrodes were dried in air and further heated at 150 °C for 1 h under a N_2_ gas flow. The electrochemical measurements were performed in a three-electrode configuration system using a CHI 760E electrochemical workstation (CH Instruments, China), including the as-prepared FTO working electrodes (with an active area of 1.0 cm^2^), Pt foil as the counter electrode and saturated calomel electrode (SCE) as the reference electrode. 0.5 M Na_2_SO_4_ aqueous solution was used as the electrolyte.

## 3. Results and discussion

**Fig. 2:**
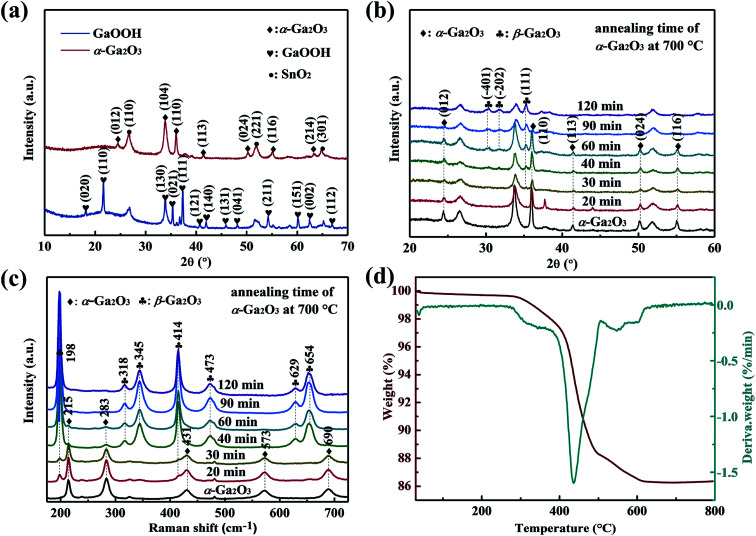
(a) XRD patterns of as-synthesized GaOOH and α-Ga_2_O_3_ NRAs. (b) XRD patterns and (c) UV Raman spectra of α-Ga_2_O_3_ NRAs annealed in air at 700 °C for various times. (d) TG/DTG curve of the as-prepared GaOOH NRAs precursor by heating from room temperature to 800 °C in air.

Fig. 2(a) shows the XRD patterns of as-synthesized GaOOH and α-Ga_2_O_3_ NRAs. All the peaks can be indexed to the orthorhombic GaOOH phase (JCPDS no. 06-0180) except the diffraction peak of the SnO_2_ seed layer. After annealing GaOOH at 400 °C for 4 h, the observed diffraction peaks occur at new locations, indicating that the GaOOH is completely converted into α-Ga_2_O_3_ of the corundum structure (JCPDS no. 06-0503).^50^ The phase transition of α-Ga_2_O_3_ at 700 °C for various times was also analyzed by XRD, and the corresponding results are shown in Fig. 2(b). With α-Ga_2_O_3_ annealed at 700 °C for 30 min, a diffraction peak corresponding to the (111) plane attributed to monoclinic β-Ga_2_O_3_ is detected, and it becomes stronger with the further increase of annealing time. Ga_2_O_3_ with different phase structures can be obtained during annealing for 30–90 min. No diffraction peak assigned to α-Ga_2_O_3_ is observed through annealing for 120 min, suggesting that the α-Ga_2_O_3_ is totally transformed into β-Ga_2_O_3_ at this point.

UV Raman spectroscopy was also used to monitor the α to β phase transformation of Ga_2_O_3_. As shown in Fig. 2(c), it is worth noting that the typical characteristic Raman bands of β-Ga_2_O_3_ at 198 cm^−1^ and 414 cm^−1^ can be clearly observed after annealing α-Ga_2_O_3_ at 700 °C for 20 min, in addition to the existing Raman bands of α-Ga_2_O_3_, indicating the formation of β-Ga_2_O_3_. However, only Raman bands at 215 cm^−1^ and 283 cm^−1^ attributed to α-Ga_2_O_3_ are detected with annealing time to 60 min, and both disappeared at 90 min. The result is unsynchronized with that revealed by the XRD patterns, which can be attributed to the strong sensitivity of UV Raman spectroscopy to the surface region, and XRD mainly reflects the bulk information of materials.^47^ Based on the above results, we suggest that the samples annealed for 20–90 min are α/β-Ga_2_O_3_ phase junction. In the following sections, the α-Ga_2_O_3_ NRAs annealed at 700 °C for various time will be labeled as Ga_2_O_3_-20 (annealed for 20 min), Ga_2_O_3_-30 (annealed for 30 min), Ga_2_O_3_-40 (annealed for 40 min), Ga_2_O_3_-60 (annealed for 60 min), Ga_2_O_3_-90 (annealed for 90 min) and β-Ga_2_O_3_ NRAs (annealed for 120 min), respectively.

The TG/DTG curve of the as-prepared GaOOH NRAs precursor by heating from room temperature to 800 °C in air atmosphere is shown in Fig. 2(d). A major weight loss of 11.2% can be noticed in the temperature range of approximately 260–480 °C, with the fastest weight loss rate occurring at 438 °C, which is attributed to transformation of GaOOH into α-Ga_2_O_3_ by thermal dehydration. A weak weight loss of 2% is also noted at the range of 500–630 °C, indicating the conversion of α-Ga_2_O_3_ to β-Ga_2_O_3_. With further prolonged heating up to 800 °C, there is no weight loss.

**Fig. 3:**
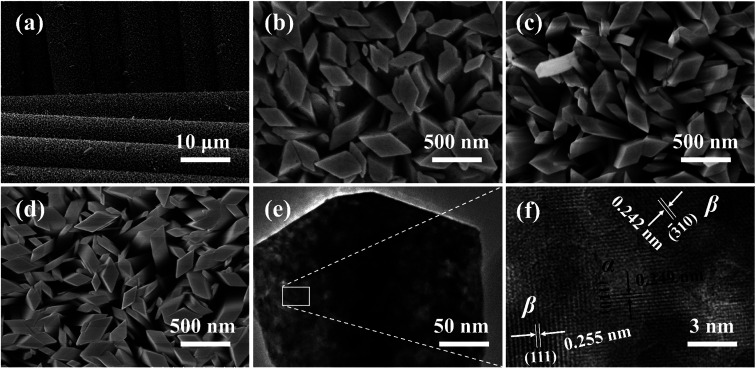
(a) SEM images of α-Ga_2_O_3_ NRAs. High-magnification SEM images of (b) α-Ga_2_O_3_ NRAs, (c) Ga_2_O_3_-60 NRAs, (d) β-Ga_2_O_3_ NRAs. (e) Low and high resolution TEM images of (f).

A typical SEM image of as-synthesized α-Ga_2_O_3_ NRAs, as presented in Fig. 3(a), which reveals the uniform and dense growth of the sample on each fiber rod. High-magnification SEM images of α-Ga_2_O_3_ NRAs and other Ga_2_O_3_ NRAs obtained by annealing (Ga_2_O_3_-60 and β-Ga_2_O_3_) are also shown in Fig. 3(b–d), respectively. Further revealing that the diameter of all nanorods ranged from 100 to 400 nm and the tips are all diamond-shaped. The annealing process has not significantly changed the morphology of Ga_2_O_3_ NRAs.^51^Fig. 3(e and f) shows the low and high resolution TEM images of the Ga_2_O_3_-60 NRAs. Different lattice fringes are observed in here. The lattice-spacing value of 0.249 nm matches the (110) planes of α-Ga_2_O_3_, while the lattice-spacing values of 0.242 nm and 0.255 nm are ascribed to the (−310) and (111) planes of β-Ga_2_O_3_. This result clearly demonstrates the formation of α/β heterophase junctions in Ga_2_O_3_-60 NRAs, supporting the previous analysis of XRD and UV Raman spectroscopy.

**Fig. 4:**
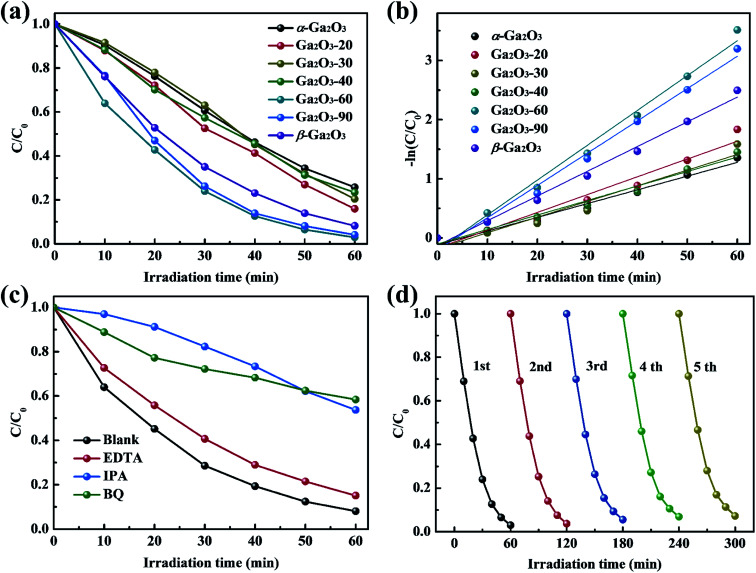
(a) Photocatalytic degradation of RhB in the presence of different Ga_2_O_3_ NRAs and (b) corresponding kinetic linear simulation curves. (c) Effect of trapping agents on the photocatalytic degradation of RhB over the Ga_2_O_3_-60 NRAs. (d) Photocatalytic stability test of the Ga_2_O_3_-60 NRAs in recycling reactions.

The photocatalytic activities of Ga_2_O_3_ NRAs were evaluated by the degradation of RhB aqueous solution under UV light irradiation. A typical UV-vis absorption spectrum of RhB aqueous solution during photocatalytic degradation process in the presence of the Ga_2_O_3_ NRAs is shown in Fig. S1.† The decrease of the characteristic peak at 554 nm during illumination suggests RhB decomposition.^1,43^Fig. 4(a) and S2† reveals the comparison of photocatalytic activities of different Ga_2_O_3_ NRAs. Among them, the Ga_2_O_3_-60 NRAs exhibits the best photocatalytic with a degradation rate of 97%, which can be attributed to the α/β-Ga_2_O_3_ phase junction promoting the separation of photogenerated electrons and holes.^47^ Furthermore, the photocatalytic degradation process of these Ga_2_O_3_ NRAs were fitted using the first-order kinetic curve according to the Langmuir–Hinshelwood model.^32,52^ As shown in Fig. 4(b), the value of the reaction rate constant (*K*) are estimated to be 0.0232, 0.0301, 0.0265, 0.0245, 0.0589, 0.0546 and 0.0418 min^−1^, corresponding to the α-Ga_2_O_3_, Ga_2_O_3_-20, Ga_2_O_3_-30, Ga_2_O_3_-40, Ga_2_O_3_-60, Ga_2_O_3_-90 and β-Ga_2_O_3_ NRAs, respectively.

Generally, a series of photogenerated reactive species, such as h^+^, ˙O_2_^−^ and ˙OH, are involved in the photocatalytic process.^4,53^ To reveal the main reactive species responsible for the degradation of RhB solution by the Ga_2_O_3_-60 NRAs, radical trapping experiments were performed by adding EDTA (ethylenediamine tetraacetic acid, h^+^ trapping agents), IPA (isopropyl alcohol, ˙OH trapping agents) and BQ (benzoquinone, ˙O_2_^−^ trapping agents), respectively. As shown in Fig. 4(c), the photocatalytic activity of the Ga_2_O_3_-60 NRAs is affected slightly with the addition of EDTA, indicating that h^+^ is not the main factor in this system. In contrast, the introduction of IPA or BQ greatly suppressed the photocatalytic activity of the Ga_2_O_3_-60 NRAs, indicating that ˙OH and ˙O_2_^−^ acted as dominating reactive species in the reaction system.

Moreover, the cycling stability of the Ga_2_O_3_-60 NRAs was evaluated by conducting five consecutive cycle degradation experiments. As shown in Fig. 4(d), the degradation ratio of RhB is not obviously reduced during the repeated experiments, indicating the remarkable stability of the Ga_2_O_3_-60 NRAs. XRD patterns (Fig. S3†) also indicates that no structural difference can be observed between the Ga_2_O_3_-60 NRAs before and after photocatalytic degradation of RhB solution.

**Fig. 5:**
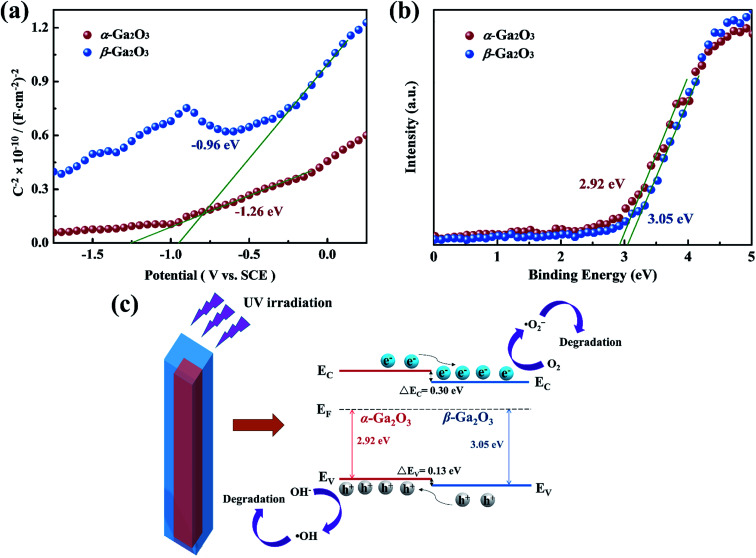
(a) Mott–Schottky curves of α-Ga_2_O_3_ NRAs and β-Ga_2_O_3_ NRAs electrodes measured in 0.5 M Na_2_SO_4_ solution. (b) XPS valence band spectra of α-Ga_2_O_3_ NRAs and β-Ga_2_O_3_ NRAs. (c) Schematic illustration of photocatalytic reaction process and charge separation transfer of α/β-Ga_2_O_3_ phase junction under UV light irradiation.

For the proposed photocatalytic degradation mechanism of the system, the band structures of α-Ga_2_O_3_ and β-Ga_2_O_3_ were characterized by Mott–Schottky measurements and XPS. As shown in Fig. 5(a and b), the flat band potential of α-Ga_2_O_3_ is calculated to be −1.26 eV (*vs.* SCE), which is more negative than the −0.96 eV (*vs.* SCE) of β-Ga_2_O_3_, and the valence band potential of β-Ga_2_O_3_ is 3.05 eV, which is more positive than the 2.92 eV of α-Ga_2_O_3_. Further combined with the band gap of Ga_2_O_3_ reported in our previous work,^51^ a schematic illustration of photocatalytic reaction process and charge separation transfer of α/β-Ga_2_O_3_ phase junction under UV light irradiation is shown in Fig. 5(c). Under UV light irradiation, the internal electric field of the α/β-Ga_2_O_3_ phase junction could drive the photogenerated charge transfer, promoting the photogenerated electrons transfer from the α phase to the β phase, and the photogenerated holes transfer from the β phase to the α phase. Following that, the photogenerated electrons react with O_2_ to generate ˙O_2_^−^ and the photogenerated holes oxidize OH^−^ to ˙OH, which together involve in RhB degradation. Efficient charge separation inhibits their recombination, resulting in improved photocatalytic degradation performance.^47,49^ In addition, it should be mentioned that phase junction can form on both the surface of Ga_2_O_3_ and in the bulk. Although almost no phase junctions were observed on the surface of the Ga_2_O_3_-60 NRAs, they still function as charge separation centers in the bulk. The separated carriers eventually diffuse to the surface of the sample to participate in the photocatalytic reaction.^4^ On the other hand, for Ga_2_O_3_ as a photocatalytic degradation material, the performance of the β phase is generally better than that of the α phase,^7,43^ which is confirmed in Fig. 4(a) of this research. Therefore, in addition to the efficient charge separation due to the phase junction in the bulk, the excellent photocatalytic activity of the Ga_2_O_3_-60 NRAs is also derived from the inherently high activity of the β surface phase. Interestingly, the Ga_2_O_3_-20, Ga_2_O_3_-30 and Ga_2_O_3_-40 NRAs did not exhibit excellent photocatalytic activity despite the formation of phase junctions on various surfaces. The phase transformation of Ga_2_O_3_ is a surface-preferred process, which is accompanied by the formation of defects.^54^ These defects may become the recombination center of photogenerated electron–hole pairs, reducing the number of efficient carriers on the surface.^55^ As a result, the photocatalytic activity of the initially annealed Ga_2_O_3_ phase junction NRAs was not satisfactory.

**Fig. 6:**
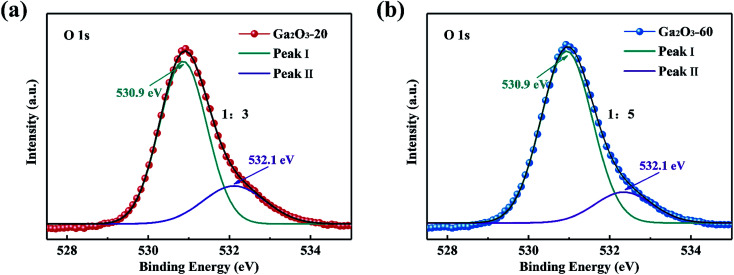
O 1s XPS spectra of (a) Ga_2_O_3_-20 NRAs and (b) Ga_2_O_3_-60 NRAs.

To obtain more insight into the effect of defects on photocatalytic activity, the Ga_2_O_3_-20 NRAs and Ga_2_O_3_-60 NRAs were selected as typical samples for XPS analysis. As shown in Fig. 6, the O 1s spectra could be divided into two peaks: I and II, representing lattice oxygen ions and oxygen ions in the oxygen vacancies region, respectively.^28^ The peak ratio (II/I) of the Ga_2_O_3_-20 NRAs is 1/3, which is higher than that of the Ga_2_O_3_-60 NRAs (1/5), indicating the presence of more oxygen vacancies. Obviously, the Ga_2_O_3_-20 NRAs exhibits poor photocatalytic activity due to the existence of abundant oxygen vacancy defects.

The comparison of the photocatalytic degradation activity of α/β-Ga_2_O_3_ phase junction NRAs in this work and other previously reported Ga_2_O_3_ related materials is listed in Table 1. Although the comparison of photocatalytic activity is not absolutely reasonable due to the different light source conditions and pollutants used in each experiment, the photocatalytic activity of α/β-Ga_2_O_3_ phase junction NRAs in this work is significantly superior to almost all previous reports on Ga_2_O_3_ related materials. This method has realized the large-area growth of α/β-Ga_2_O_3_ phase junction NRAs on the flexible glass fiber fabric and obviously improved its photocatalytic performance, which is of great significance in the future research in the field of photocatalysis.

**Table 1:** Comparison of the photocatalytic activity of a selection of previously reported Ga_2_O_3_ related materials and this work

Photocatalyst, concentration (mg L^−1^)	Pollutants, concentration (mol L^−1^)	Light source	Degradation after 60 min	Reference
α-Ga_2_O_3_ nanoplates, 90	RhB, 0.45 × 10^−5^	AM 1.5 solar simulator	53%	1
α-Ga_2_O_3_ nanoparticles, 400	TC, 5.6 × 10^−5^	30 W UV lamp	85%	5
α-Ga_2_O_3_ nanorods, 1000	RhB, 0.84 × 10^−5^	300 W Hg lamp	62%	33
β-Ga_2_O_3_ nanorods, 1000	RhB, 2 × 10^−5^	150 W xenon lamp	39%	36
β-Ga_2_O_3_ microspheres, 1000	RhB, 2 × 10^−5^	150 W xenon lamp	60%	37
Ga_2_O_3_ sheet, 500	CR, 2.15 × 10^−5^	30 W UV lamp	33%	38
β-Ga_2_O_3_ nanorods, 1000	RhB, 2 × 10^−4^	1000 W UV lamp	38%	43
TiO_2_–Ga_2_O_3_ heterojunctions	MO, 1.8 × 10^−5^	30 W UV lamp	83%	45
α/β-Ga_2_O_3_ NRAs, 200	RhB, 2 × 10^−5^	10 W UV lamp	97%	This work

## 4. Conclusions

In summary, large-area Ga_2_O_3_ NRAs with controllable α/β phase junction were firstly prepared *in situ* on a flexible glass fiber fabric by a facile and environmentally friendly three-step method. Photocatalytic degradation experiments showed that the α/β-Ga_2_O_3_ phase junction NRAs synthesized by annealing α-Ga_2_O_3_ NRAs at 700 °C for 60 min exhibited remarkable performance for RhB, with a degradation rate of 97% in 60 min under UV light. The enhanced photocatalytic activity can be attributed to the unique phase junction promoting efficient charge separation and inhibiting the recombination of photogenerated electron–hole pairs. Additionally, the glass fiber fabric can realize large-area growth of the Ga_2_O_3_ NRAs, effectively solve the trouble of difficult recovery and reuse of photocatalysts, as well as the insufficient absorption of light. A facile environmentally friendly and inexpensive synthesis route will open new avenues for the development of efficient photocatalysts.

## Conflicts of interest

There are no conflicts to declare.

## Supplementary Material

RA-010-D0RA01461C-s001
