# Supplementary material for: Phase junction enhanced photocatalytic activity of Ga2O3 nanorod arrays on flexible glass fiber fabric
Source: RSC Adv. 2020 Mar 20;10(19):11499–506. doi: 10.1039/d0ra01461c (PMC9050499; doi:10.1039/d0ra01461c)
Supplement: RA-010-D0RA01461C-s001 [file RA-010-D0RA01461C-s001.pdf]

Electronic Supplementary Material (ESI) for RSC Advances.
This journal is © The Royal Society of Chemistry 2020

Supplementary Material

Phase junction enhanced photocatalytic activity of Ga_2O_3 nanorod arrays on flexible glass fiber fabric

Hanlin Sun[a], Liying Zhang[a], Jingyan Yu[a], Shunli Wang[a*], Daoyou Guo[a], Chaorong Li[a], Fengmin Wu[a], Aiping Liu[a], Peigang Li[b], Weihua Tang[b]

[a] Key Laboratory of Optical Field Manipulation of Zhejiang Province&Center for Optoelectronics Materials and Devices, Department of Physics, Zhejiang Sci-Tech University Hangzhou, 310018, China.

[b] State Key Laboratory of Information Photonics and Optical Communications & Information Functional Materials and Devices, School of Science, Beijing University of Posts and Telecommunications, Beijing 100876, China.

* Corresponding author e-mail addresses: **slwang@zstu.edu.cn**

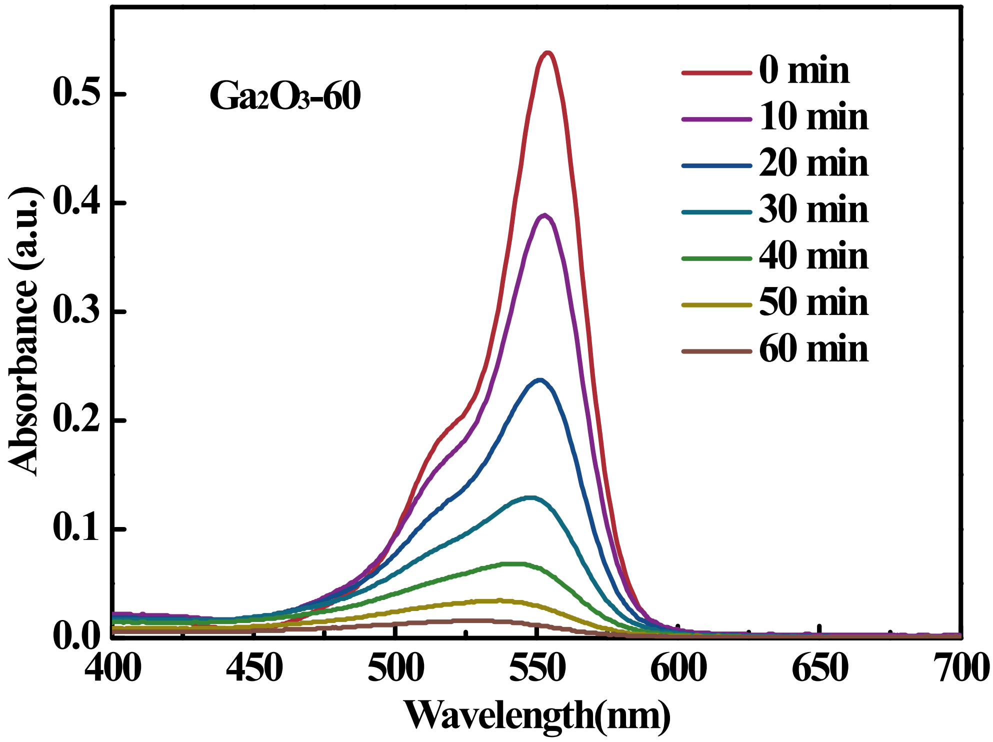

Fig. S1. UV-vis absorption spectrum of RhB solution in the presence of the Ga_2O_3-60 NRAs under UV light irradiation.

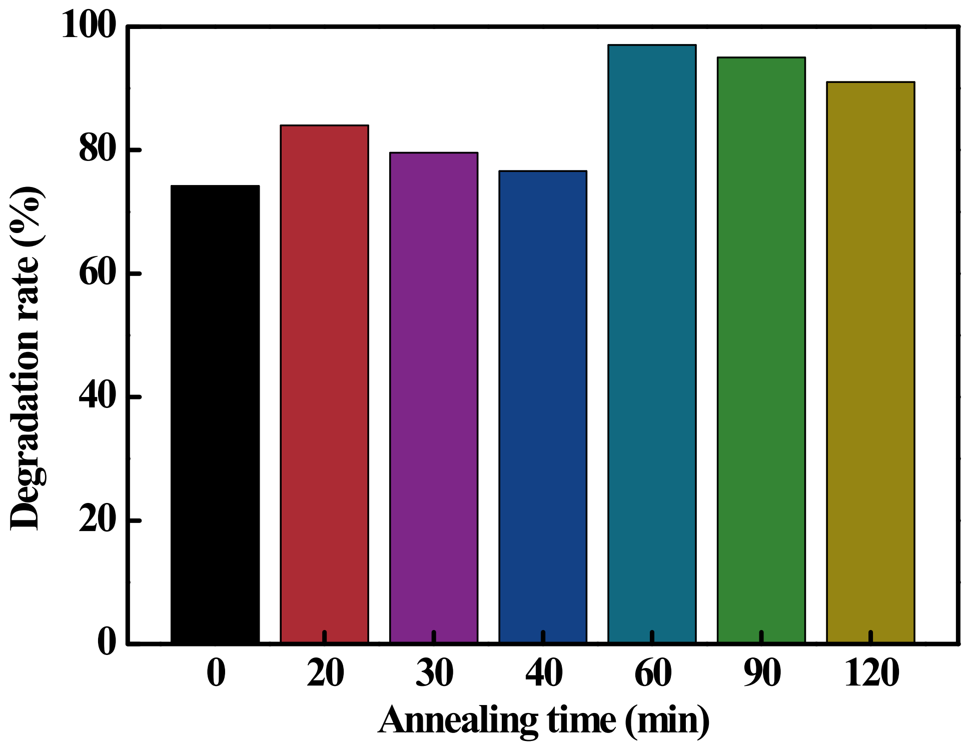

Fig. S2. Histogram comparison of photocatalytic degradation of RhB solution in the presence of different Ga_2O_3 NRAs (α-Ga_2O_3 annealing at 700 °C for different times) after 60 min of UV light irradiation.

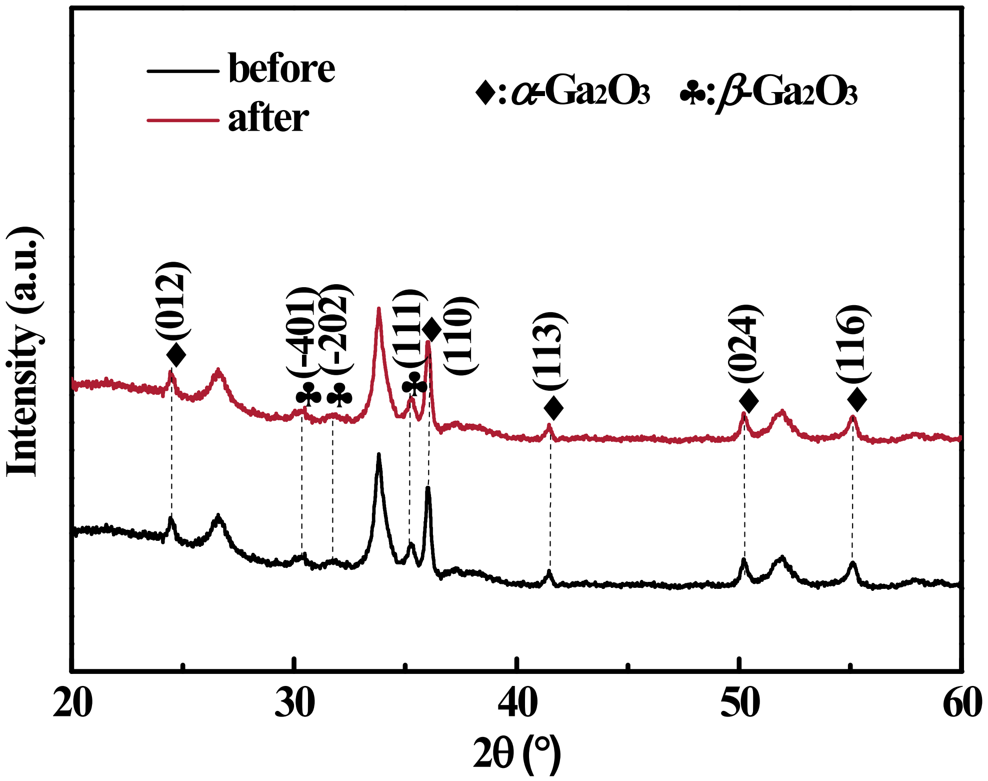

Fig. S3. XRD patterns of the Ga_2O_3-60 NRAs before and after photocatalytic degradation of RhB solution.
